# Implicit Motor Imagery and the Lateral Occipitotemporal Cortex: Hints for Tailoring Non-Invasive Brain Stimulation

**DOI:** 10.3390/ijerph17165851

**Published:** 2020-08-12

**Authors:** Massimiliano Conson, Roberta Cecere, Chiara Baiano, Francesco De Bellis, Gabriela Forgione, Isa Zappullo, Luigi Trojano

**Affiliations:** 1 Laboratory of Developmental Neuropsychology, Department of Psychology, University of Campania Luigi Vanvitelli, 81100 Caserta, Italy; roberta.cecere@hotmail.com (R.C.); chiara.baiano@unicampania.it (C.B.); gabrielaforgione@outlook.it (G.F.); isa.zappullo@unicampania.it (I.Z.); 2 Laboratory of Neuropsychology, Department of Psychology, University of Campania Luigi Vanvitelli, 81100 Caserta, Italy; franc.debellis@gmail.com (F.D.B.); luigi.trojano@unicampania.it (L.T.)

**Keywords:** rTMS, hand laterality judgment, human hand, visual areas, compensation

## Abstract

Background: Recent evidence has converged in showing that the lateral occipitotemporal cortex is over-recruited during implicit motor imagery in elderly and in patients with neurodegenerative disorders, such as Parkinson’s disease. These data suggest that when automatically imaging movements, individuals exploit neural resources in the visual areas to compensate for the decline in activating motor representations. Thus, the occipitotemporal cortex could represent a cortical target of non-invasive brain stimulation combined with cognitive training to enhance motor imagery performance. Here, we aimed at shedding light on the role of the left and right lateral occipitotemporal cortex in implicit motor imagery. Methods: We applied online, high-frequency, repetitive transcranial magnetic stimulation (rTMS) over the left and right lateral occipitotemporal cortex while healthy right-handers judged the laterality of hand images. Results: With respect to the sham condition, left hemisphere stimulation specifically reduced accuracy in judging the laterality of right-hand images. Instead, the hallmark of motor simulation, i.e., the biomechanical effect, was never influenced by rTMS. Conclusions: The lateral occipitotemporal cortex seems to be involved in mental representation of the dominant hand, at least in right-handers, but not in reactivating sensorimotor information during simulation. These findings provide useful hints for developing combined brain stimulation and behavioural trainings to improve motor imagery.

## 1. Introduction

An impairment of motor imagery, i.e., of the ability to mentally simulate movements, has been described in patients with severe motor disorders as locked-in syndrome [1,2], amyotrophic lateral sclerosis [3], and spinal cord injury [4], and in also in patients with neurodegeneration, such as Parkinson’s disease [5,6,7,8] and Alzheimer’s disease [9]. However, healthy aging also usually impacts motor imagery [10,11,12,13,14].

In both pathological and physiological brain conditions, the modifications of motor imagery performance are related to neurofunctional changes, mainly implying the over-recruitment of occipitotemporal areas [7,8,9,14]. Interestingly, younger elders able to perform a motor imagery task without differences compared to younger persons show an over-recruitment of the occipitotemporal cortex—the stronger the recruitment of these regions, the better the behavioural performance [13].

These data suggest that brain-damaged patients and elders tend to adopt compensatory strategies to deal with motor imagery tasks by exploiting additional neural resources in the visual areas to compensate for the decline in activating motor representations. In particular, the lateral division of the occipitotemporal cortex [7,8,9] seems to represent a good candidate for targeting non-invasive brain stimulation in combination with cognitive training to enhance motor imagery performance. For this purpose, it is crucial to clarify the exact role of this cortical region in motor imagery. Indeed, in recent years, growing data in healthy brains has demonstrated that the lateral occipitotemporal cortex is specialized in the visual perception of human bodies and of non-facial body parts [15,16], but the nature of the involvement of the lateral occipitotemporal cortex in motor imagery remains unclear.

One key point for addressing this issue relates to the distinction between two main kinds of motor imagery, i.e., explicit and implicit motor imagery. During explicit motor imagery tasks, participants are directly required to imagine themselves performing specific actions, while implicit motor imagery tasks require participants to make judgments on visual stimuli that automatically (and implicitly) activate the mental simulation of actions [17,18]. Compensatory activities of the lateral occipitotemporal cortex in motor imagery mainly refers to studies on implicit motor imagery [7,8,9,13], using different versions of the hand laterality judgment [19,20].

The hand laterality judgment is the most used task of implicit motor imagery. It requires participants to decide whether a visually presented stimulus in a given angular orientation portrays a left or a right hand [19,20]. Both classical and recent studies have demonstrated that this task implies the automatic activation of mental simulation of actions (i.e., motor imagery) [19,20,21], but several studies have shown that visual imagery can be recruited too when people make hand laterality judgments [22,23,24,25,26]. A fine-grain analysis of task performance can reveal differences between visual and motor strategies. In particular, the activation of visual strategies can account for the advantage in judging the laterality of right-hand images [24,26,27], whereas the motor component of hand laterality performance is revealed by the biomechanical effect, which is the advantage for judging hand images showing medial (towards the body midline) versus lateral (away from the body midline) orientations [19,20].

In the present study, we aimed at clarifying whether the lateral occipitotemporal region’s contribution to an implicit motor imagery task is related to implementation of visual or motor strategies. To this aim, we delivered online, high-frequency, repetitive transcranial magnetic stimulation (rTMS) over the left or right lateral occipitotemporal cortex (contrasted with sham stimulation) while participants performed the classical version of the hand laterality judgment [19,20] in a within-subject experimental design.

Transcranial magnetic stimulation (TMS) uses very brief, high-intensity magnetic fields to induce currents of depolarizing neurons in defined regions of the cortex. The neural effects of TMS depend on the frequency of stimulation. When the frequency is 1 Hz or higher, the stimulation is known as repetitive TMS (rTMS). If rTMS is pulsed at a low frequency (about 1 Hz), cortical excitability generally decreases, while higher frequency rTMS, mainly between 5 and 20 Hz, can increase cortical excitability [28,29]. Notwithstanding these general principles, rTMS can up- or downregulate cortical excitability depending on other stimulation parameters, such as duration and timing relative to a given cognitive or motor task [30]. Of relevance here, in a seminal study, Urgesi et al. [31] showed that high-frequency rTMS of a specific region of the lateral occipitotemporal cortex, the extrastriate body area (EBA), impaired visual discrimination of bodily forms, whereas stimulation of the lateral premotor cortex impaired visual discrimination of bodily actions.

In the present, using rTMS has allowed us to test the role of the lateral occipitotemporal cortex in implicit motor imagery. In particular, finding that rTMS of the lateral occipitotemporal cortex mainly affects laterality judgment of right-hand images could support a prevalent role of this region in the visual strategies thought to be activated when processing one’s own dominant (right) hand [26,27]. Instead, an rTMS interference with the biomechanical effect would support the role of the lateral occipitotemporal cortex in the motor component of hand laterality judgment. These findings could help to tailor non-invasive brain stimulation to improve motor imagery, as a function of the actual kind of impairment in a brain-damaged individual, or of the specific cognitive strategy to be boosted.

## 2. Materials and Methods

### 2.1. Participants

A group of 23 right-handed, healthy university students were recruited for the experiment. To be included in the study, each subject had to report no clinical diagnosis or past history of psychiatric, neurological, or neurodevelopmental disorders, as well as no assumption of drugs or substances acting on the central nervous system. Thus, exclusion criteria were the presence of medical conditions, as well as metal in the cranium, intracardiac lines, intracorporal electronic devices, or a family history of epilepsy for safety reasons [32]. Three out the 23 subjects did not complete the full experimental protocol; thus, 20 participants (10 females, age range: 22–29, mean age = 24.1, SD = 2.33; 10 males, age range: 21–29, mean age = 24.46, SD = 2.01) were included in the statistical analysis.

The entire protocol was approved by the Local Ethics Committee of the Department of Psychology, University of Campania Luigi Vanvitelli (N:29/2020), and was conducted in accordance with the ethical standards of the Helsinki declaration; written informed consent was obtained from all participants before starting the experiment.

### 2.2. Neuronavigation and TMS

Online trains of TMS (each train including four 10-Hz pulses, for a duration of 400 msec) were delivered by means of a 70 mm figure-eight coil connected to a Magstim Rapid 2 stimulator (Magstim Co. Ltd., Whitland, United Kingdom), producing a maximum output of 3.5 T at the coil surface (output type: biphasic; pulse width: 400 msec). In keeping with safety recommendations [32,33], two consecutive stimulation trains were separated by at least 4.5 s, and the total number of pulses per session was 512. Stimulation intensity (ranging from 44% to 62% of the maximum stimulator output) was set at 60% of the individual motor threshold, in order to reduce uncomfortable sensations possibly associated with high-intensity stimulation [32,33]. Participants’ motor threshold was established as the lowest stimulation intensity applied over the M1 capable of evoking a visible contraction in the relaxed left hand on at least four out of eight consecutive stimulations.

**Figure 1:**
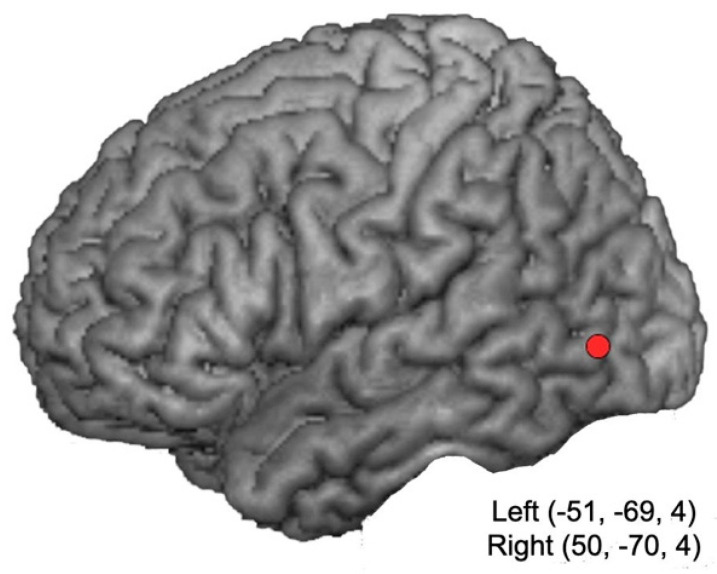
Stimulation sites marked on estimated MRI slices. The mean Talairach coordinates fell within the posterior part of the middle temporal gyrus (Brodmann’s area 37) for the left and right lateral occipitotemporal cortex.

The brain targets and the scalp stimulation sites corresponded to the left and right lateral occipitotemporal cortex [16,31], which were localized by means of the Softaxic Optic (EMS) neuronavigation system (E.M.S. srl Company, Italy). Neuronavigation was carried out on estimated MRI stereotaxic templates of the participants’ brains, based on a sample of 65 scalp points digitized by means of a Polaris Vicra digitizer (Northern Digital Inc., Waterloo, ON, Canada). The mean Talairach coordinates of brain targets were as follows: right hemisphere: *x* = 50, *y* = −70, *z* = 4; left hemisphere: *x* = −50, *y* = −70, *z* = 4 (Figure 1). In the sham condition, the coil was placed at an angle of 90° on the vertex, resting on the scalp with only one edge, so that the coil focus was directed away from participant head. Coil positioning on the stimulation sites was checked online by means of the neuronavigator system during the entire experimental session. In all conditions, a mechanical arm fixed to a tripod held the coil.

### 2.3. Experimental Task and Procedure

**Figure 2:**
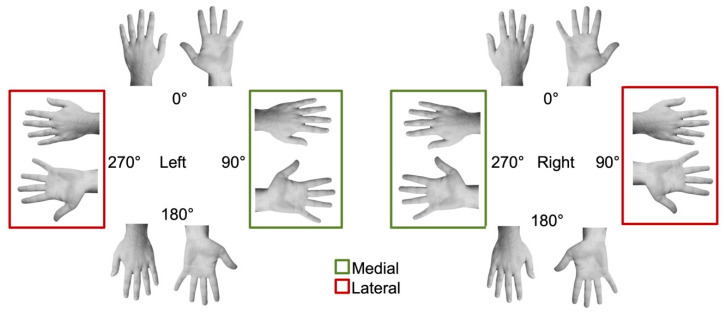
Examples of stimuli used in the hand laterality judgment. In particular, male hands viewed from the dorsum and palm are displayed. Hand stimuli taken into account to compute the biomechanical effect are highlighted: hand images showing medial (green square) versus lateral (red square) positions.

The experimental stimuli consisted of greyscale pictures of a male or female hand portrayed from the back and the palm (Figure 2). Participants had to decide whether the presented hand was the left or right. Male participants were presented with male hands, and female participants with female hands.

Participants provided their left/right judgments while sitting on a comfortable chair with the head on a chinrest, facing the monitor at a distance of about 65 cm. They kept their feet resting on a foot keyboard and held their hands on their thighs, palms facing down. Task instructions required to respond as fast and accurately as possible by pressing left or right keys on a foot pedal (X-Key PS2; P.I. Engineering, Williamston, MI, United States), according to the laterality of the hand stimulus.

**Figure 3:**
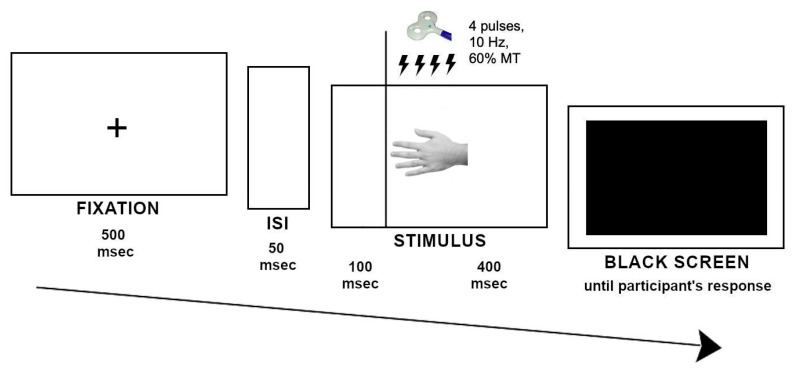
Sequence of the events in the experimental trials.

Each trial started with a fixation point (a cross) presented in the center of a blank screen for 500 msec, followed by a blank screen (50 msec). rTMS train was delivered 100 msec after the presentation of the stimulus, which lasted for 500 msec, followed by a black screen representing the “go” signal for providing the response (Figure 3). Our choice to deliver the TMS trains 100 msec after stimulus onset was based on previous studies demonstrating that neurophysiological responses of the lateral occipitotemporal cortex to the presentation of bodily stimuli can be recorded at 100–200 msec after the stimulus onset [34,35].

Three sessions in separate weeks were conducted, in which online rTMS was delivered over the right and left lateral occipitotemporal cortex or orthogonally to the vertex (sham condition). The order of stimulation conditions was counterbalanced across subjects. Before starting each of the three stimulation sessions, a training session was conducted by presenting 16 trials per task.

Stimulus presentation, randomization, and TMS triggering, as well as data collection (accuracy and reaction times (RTs)) were controlled by Superlab 4.0 software (Cedrus Corporation, San Pedro, CA, United States).

### 2.4. Statistical Analysis

Following available literature (e.g., [36,37]), trails outside the range of 500–3500 msec were discarded from the analysis. On both accuracy and RTs (msec) data, two separate ANOVAs were performed. First, a 3 × 2 × 2 × 4 repeated-measures ANOVA was carried out, with stimulation condition (right and left hemisphere, and sham), hand view (dorsum and palm), hand laterality (left and right), and orientation (0°, 90°, 180°, 270°) as within-subject factors. Second, we specifically tested whether the biomechanical effect could interact with stimulation condition. The biomechanical effect was computed by comparing responses to images showing lateral hand positions (270° oriented left hand and 90° oriented right hand) with responses to images showing medial hand positions (90° oriented left hand and 270° oriented right hand) separately for backs and palms (Figure 2). Then, a 3 × 2 × 2 repeated-measures ANOVA was performed, with stimulation condition (right and left hemisphere and sham), view (back and palm), and hand position (medial and lateral) as within-subject factors. The Greenhouse–Geisser correction was applied when Mauchly’s test was significant, indicating that the assumption of sphericity had been violated, but uncorrected degrees of freedom were reported for transparency. When required, post-hoc Bonferroni’s corrected pairwise comparisons were performed.

Data analysis was executed with SPSS v.19 software (IBM Corp., Armonk, NY, USA). Raw data are available upon request from the first author.

## 3. Results

In total, 3840 trials were recorded, and 98.3% of the total number of trials were included in the analysis (removed trials = 67/3840; total loss of trials = 1.7%).

### 3.1. Hand Laterality and Spatial Orientation

#### 3.1.1. Accuracy

**Figure 4:**
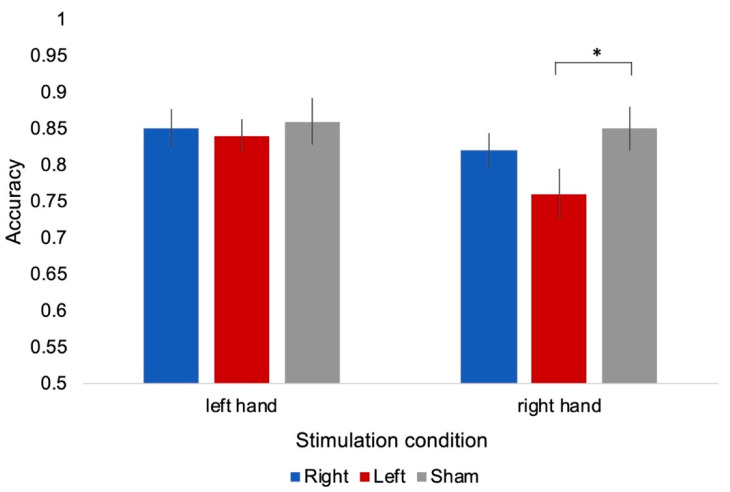
Mean accuracy (bars are standard error of measurement, SEM) as a function of stimulation condition (right, left, and sham) and laterality of hand stimuli. * Bonferroni post-hoc comparisons, significant at *p* = 0.045.

Mean accuracy is reported in Table 1. Results of the ANOVA showed a significant main effect of hand laterality (F(1,19) = 5.787, *p* = 0.026, partial η^2^ = 0.233), with lower accuracy when judging right-hand (mean = 0.81; SEM = 0.025) than left-hand stimuli (mean = 0.85; SEM = 0.022), as well as a an effect of stimulus orientation (F(3,57) = 18.453, *p* = 0.0001, partial η^2^ = 0.493), with significantly lower accuracy when participants judged hands at 180° (mean = 0.71, SEM = 0.032) than in all the other conditions (0°: mean = 0.87, SEM = 0.024; 90°: mean = 0.87, SEM = 0.025; 270°: mean = 0.86, SEM = 0.028; all: *p* = 0.001). Importantly, we found a significant interaction between stimulation condition and hand laterality (F(2,38) = 3.639, *p* = 0.036, partial η^2^ = 0.161); post-hoc comparisons showed a significantly lower accuracy when judging right hands in the left stimulation than in the sham condition (*p* = 0.045), while no significant differences were found between left and right stimulation conditions (*p* > 0.05). Moreover, no significant difference was found between the three stimulation conditions when judging the left-hand stimuli (all *p* > 0.05; Figure 4). Finally, results showed a significant interaction between stimulus view and orientation (F(3,57) = 3.619, *p* = 0.033, partial η^2^ = 0.160), due to lower accuracy for 180° stimuli with respect to the other three orientations, although the difference was significant for hands viewed from back (all *p* = 0.0001), but not from palm (all *p* > 0.05). No other main effect or interaction was significant (all *p* > 0.05).

#### 3.1.2. RTs

Mean RTs are reported in Table 2. Results showed significant main effects of view (F(1,19) = 8.177, *p* = 0.010, partial η^2^ = 0.301), due to slower RTs for palms (mean = 1311, SEM = 67.1) than backs (mean = 1225, SEM = 71.6), and of hand orientation (F(1.43,27.29) = 18.125, *p* = 0.0001, partial η^2^ = 0.488), due to slower RTs for 180° hands (mean = 1474, SEM = 88.6) than for the other orientations (0°: mean = 1171, SEM = 65.5; 90°: mean = 1218, SEM = 64.9; 270°: mean = 1211, SEM = 72.1). Moreover, we found a significant interaction between stimulus view and orientation (F(3,57) = 14.851, *p* = 0.0001, partial η^2^ = 0.439), as for both backs and palms RTs were slower for 180° hands than for the other orientations. However, the differences were always significant for backs (all *p* < 0.002), while for palms only 180° hands vs. both 90° and 270° comparisons were significant (*p* < 0.043). Finally, we found a significant interaction between hand laterality and orientation (F(3,57) = 8.318, *p* = 0.0001, partial η^2^ = 0.304), with faster RTs for the left (mean = 1127, SEM = 57.6) than the right hand (mean = 1309, SEM = 81.9) at 90° (*p* = 0.004), and the reverse pattern at 270° (left hand: mean = 1280, SEM = 74.9; right hand: mean = 1140, SEM = 75.4; *p* = 0.004), consistent with the classical effect of biomechanical constraints on the mental rotation of hands [19]. No other main effect or interaction was significant (all *p* > 0.05).

### 3.2. Biomechanical Effect

#### 3.2.1. Accuracy

Mean accuracy is reported in Table 3. Results did not show significant main effects (*p* > 0.05), but a trend towards significance of the stimulation condition (F(2,38) = 3.026, *p* = 0.078, partial η^2^ = 0.137), with lower accuracy in the left (mean = 0.83; SEM = 0.033) than in the right (mean = 0.89; SEM = 0.023) stimulation condition and sham (mean = 0.88; SEM = 0.031), as well as a significance trend for hand view (F(1,19) = 3.736, *p* = 0.068, partial η^2^ = 0.164), with lower accuracy for palm (mean = 0.84; SEM = 0.03) than dorsum (mean = 0.89; SEM = 0.028). No interaction was statistically significant (all *p* > 0.05).

#### 3.2.2. RTs

Mean RTs are reported in Table 4. Results showed significant main effects of view (F(1,19) = 13.299, *p* = 0.002, partial η^2^ = 0.412), with slower RTs to the palm (mean = 1269; SEM = 64.5) than the dorsum (mean = 1159; SEM = 74.2), and of hand position (F(1,19) = 18.495, *p* = 0.0001, partial η^2^ = 0.493), with slower RTs to hands in the lateral (mean = 1294; SEM = 76.1) than in the medial position (mean = 1134; SEM = 64.2), confirming the biomechanical effect [19]. No interaction was statistically significant (all *p* > 0.05).

## 4. Discussion

We tested the role of the lateral occipitotemporal cortex in implicit motor imagery by delivering rTMS while participants performed the classical version of the hand laterality judgment [19,20]. The results demonstrated a specific role of the left occipitotemporal cortex in judging the laterality of hand images, since participants were significantly less accurate when judging the laterality of right-hand images in the left stimulation condition than in the sham condition, whereas no significant differences were found between left and right stimulation conditions, nor between the three stimulation conditions when participants had to judge left-hand images. Importantly, the biomechanical effect, a marker of motor simulation in hand laterality judgment, was never influenced by rTMS over the lateral occipitotemporal cortex.

Studies on healthy participants demonstrating the involvement of the lateral occipitotemporal cortex in implicit motor imagery have used adapted versions of the hand laterality judgment, requiring participants to decide the laterality of their own (vs. others’) hand images presented in different spatial orientations [27,38]. For instance, in a functional magnetic resonance (fMR) study, Ferri et al. [27] found stronger right occipitotemporal cortex activation when participants judged the laterality of images portraying their own dominant (right) hand. In an rTMS study, though, De Bellis et al. [38] reported faster responses for others’ hands while stimulating the right occipitotemporal cortex, and an advantage on self-hands during stimulation of the left occipitotemporal cortex. These data might suggest that the lateral occipitotemporal cortex is implied in hand laterality judgment when the participants have to deal with images of their own bodies. Instead, the present findings show that judging an image of one’s own body is not a prerequisite to recruit the lateral occipitotemporal cortex during hand laterality judgment. Indeed, here we presented participants with hand stimuli not belonging to themselves, and task instructions did not make any reference to the need of imagining one’s own body to perform the task. Thus, as rTMS selectively influenced the judgment of right-hand images, and did not modulate the biomechanical effect, the findings from the present study would support a main role of the lateral occipitotemporal region in the visual strategies activated when mentally rotating dominant (right) hands [26,27,39].

The lateral occipitotemporal cortex has been considered a high-level, category-specific object recognition area that is specialized for the visual analysis of human bodies and body parts, similar to other category-specific areas in the visual cortex (as face-selective areas) [15,16,40]. Two hand-sensitive subregions have been identified in the left occipitotemporal cortex: the lateral occipital sulcus and the EBA [15,41]. In the present study, the stimulation sites of left and right lateral occipitotemporal cortex corresponded to left and right EBA, respectively [16,31]. Although it is important to bear in mind that rTMS-related activation of outputs from the stimulation site could potentially disrupt processing at an adjacent, or even at the distant site [42], we could suggest that the present stimulation protocol interfered with the activity of EBA playing its key role in processing the body parts as hand images [38,41]. Consistently, the EBA is the functional region of the lateral occipitotemporal cortex specifically over-recruited in patients with Parkinson’s disease performing the hand laterality task. Indeed, in an fMRI study, Helmich et al. [7] investigated neural correlates of implicit motor imagery in right-handed patients with Parkinson’s disease with strong right-lateralized symptoms (i.e., with predominant motor impairment on their right hand and body side). The authors manipulated the posture of the patients’ arms while they judged laterality of hand stimuli. in order to test the possible congruency advantage, i.e., the improvement in performance when current hand posture is congruent to the imagined movement. The results showed that the patients were impaired in judging right hand stimuli, corresponding to their the most affected hand, especially when depicted in biomechanically complex positions, but without the posture congruence effect. The neurofunctional data showed increased activity of the lateral occipitotemporal cortex, and in particular of the EBA, while patients judged images corresponding to the most affected hand. Since this region increased its connectivity towards the left premotor cortex for right (affected) hand images in a lateral orientation, the authors suggested that in patients with strongly lateralized symptoms, motor imagery of the most affected hand exploits additional resources in the lateral occipitotemporal cortex. These results imply a compensatory role of visual processing during motor imagery in Parkinson’s disease. Unfortunately, Helmich et al. [7] did not compare the neurofunctional activations of patients versus healthy controls; thus, the meaning of such lateral occipitotemporal cortex activation remains not fully understood. In a following rTMS study, van Nuenen et al. [8] aimed at clarifying the compensatory occipitotemporal activity in Parkinson’s disease by stimulating this region, together with the dorsal premotor cortex during the hand laterality judgment experiment used in Helmich et al. [7]. The results were not completely consistent with the previous ones, since in this experiment inhibition of the occipitotemporal cortex disrupted the posture congruence advantage without interfering with judgments on right-hand stimuli (corresponding to the most affected side) and with the biomechanical effect. Moreover, van Nuenen et al. [8] found that inhibition of the dorsal premotor cortex did not affect the patients’ posture congruence effect, whereas it modulated this effect (but not the biomechanical effect) in healthy controls. On this basis, the authors suggested that the lateral occipitotemporal cortex plays a key role in Parkinson’s disease patients, to compensate for a reduced contribution of the premotor cortex to mentally simulate movements.

The present findings on healthy individuals, showing a specific interference of rTMS on judgments of right-hand images, are strikingly consistent with Helmich et al.’s [7] data on Parkinson’s disease, while the lack of rTMS on the biomechanical effect is consistent with van Nuenen et al.’s data [8]. It is worth noting here that posture manipulation during hand laterality judgment can alter the classical way in which the participants deal with the task, even producing unexpected interference of posture manipulation upon task performance in the congruent conditions, rather than the congruence effect [36,43,44]. Moreover, manipulating body posture, as in both Helmich et al.’s [7] and Nuenen et al.’s [8] studies, elicits proprioceptive and tactile information, also affecting the activity of the lateral occipitotemporal cortex independently from the requirement to concurrently perform the hand laterality task [45,46,47]. Neuroimaging studies have demonstrated the crucial role of a functional network in the left hemisphere, involving the lateral occipitotemporal and premotor cortexes, in integrating proprioceptive, tactile, and visual information for building a multisensory representation of one’s own limbs [45,48]. Accordingly, in an rTMS study on the famous rubber hand illusion, Wold et al. [47] showed that participants misjudged their real hand’s location towards the dummy hand more often after left lateral occipitotemporal cortex stimulation than after sham rTMS stimulation.

From this perspective, our results from the classical version of the hand laterality task could reconcile data on compensatory activity of the lateral occipitotemporal cortex during implicit motor imagery, supporting the view that the left lateral occipitotemporal cortex plays a primary role in integrating different sources of information, in particular visual and proprioceptive ones [41,45,46,47,48], in building a visual representation of the dominant (right) hand, at least in right-handers. Such a contribution to action simulation would increase as the motor representation of one’s own hand became weaker, as with both pathological and physiological brain changes. However, it is worth underscoring here that in the present study, we only assessed right-handed participants, thus not allowing us to clarify whether the involvement of the left lateral occipitotemporal in representing one’s own dominant hand is specific for right-handers, or it could be found in left-handers as well. Since the available literature on implicit motor imagery has converged in suggesting that handedness affects performance on the hand laterality judgment [44,49,50], this issue should be directly tested in specific research. Moreover, the present results were gathered from a small sample size, thus further investigation is needed with a larger sample size.

Notwithstanding the above study limitations, from a translational point of view, our data support the idea that the lateral occipitotemporal cortex could represent a cortical target of non-invasive brain stimulation in persons engaged in implicit motor imagery training, also considering that non-invasive brain stimulation, as currently rTMS is applied to non-motor areas, appears to be safe with only a few, and generally mild, adverse effects [32,33,51].

Studies on patients affected by movement disorders due to stroke [52] or Parkinson’s disease [7,8,53,54] have demonstrated that facilitation of the left lateral occipitotemporal cortex activity could boost activation of a visual representation of the patients’ dominant limb, bypassing the reduced capacity to internally trigger motor imagery. Indeed, the activation of visual hand representation in the lateral occipitotemporal cortex, integrating multisensory information, could feed the premotor and parietal cortex [8,45,46,47,48] for planning the motor simulation steps of motor imagery [8,48,55]. However, it is important to bear in mind that a recent study on explicit motor imagery in elders has shown that the more the participants relied on visual information during motor imagery (with stronger occipitotemporal activation), the more inaccurate they were in judging the time needed to imagine walking a path [56]. Thus, targeting visual areas to facilitate motor imagery could represent a specific strategy in rehabilitation settings with patients who are able to exploit visual information to imagine movements. In this respect, a visually-based facilitation of motor simulation could also be fruitful in healthy participants, such as dancers or athletes, who have been proven to be able to use the visual strategy and integrate it with the motor strategy, but taking into account key parameters of the simulated actions, such as the type of movement or its complexity [57,58,59,60,61]. Finally, it is worth underlining that understanding the neural mechanisms involved in motor imagery is crucial for improvement of the brain–computer interface (BCI) and bioprosthetic technologies. In particular, methodological choices, such as training the right or left hand, providing a visual or a proprioceptive feedback while training, and exploiting the sensorimotor activity of the premotor and parietal cortex (or rather, the visual integration activity of the lateral occipitotemporal cortex) are relevant issues for improving these emerging technologies and implementing them in individuals who cannot learn using a motor imagery-based BCI, even despite extreme training [62,63].

## 5. Conclusions

In synthesis, the main results of the present study show that, with respect to the sham condition, left hemisphere stimulation reduces accuracy in judging the laterality of right-hand images. However, the hallmark of motor simulation, i.e., the biomechanical effect, was never affected by rTMS over the lateral occipitotemporal cortex. Thus, the lateral occipitotemporal cortex seems to be involved in the mental representation of the dominant hand, at least in right-handers, rather than being directly implied in reactivating sensorimotor information during simulation. These findings provide useful hints for developing combined brain stimulation and behavioural trainings to improve motor imagery.

## Figures and Tables

**Table 1:** Mean accuracy and SD, separately for hand view (dorsum and palm), orientation (0°, 90°, 180°, and 270°), laterality (left and right), and stimulation condition (left, right, and sham).

	Dorsum				Palm			
	0°	90°	180°	270°	0°	90°	180°	270°
Right hand								
Left rTMS	0.89	0.78	0.65	0.88	0.74	0.74	0.65	0.74
	0.22	0.29	0.28	0.22	0.26	0.30	0.30	0.32
Right rTMS	0.87	0.89	0.59	0.89	0.81	0.85	0.81	0.85
	0.19	0.21	0.35	0.26	0.27	0.24	0.22	0.22
Sham	0.93	0.96	0.78	0.90	0.83	0.80	0.71	0.85
	0.14	0.09	0.24	0.19	0.33	0.29	0.30	0.22
Left hand								
Left rTMS	0.91	0.90	0.68	0.86	0.89	0.90	0.73	0.86
	0.17	0.22	0.37	0.19	0.22	0.13	0.24	0.21
Right rTMS	0.87	0.90	0.65	0.95	0.94	0.93	0.73	0.84
	0.18	0.25	0.32	0.11	0.16	0.12	0.33	0.19
Sham	0.89	0.90	0.73	0.91	0.90	0.89	0.80	0.83
	0.19	0.26	0.27	0.19	0.19	0.24	0.26	0.28

**Table 2:** Mean reaction times (RTs) (msec) and SD, separately for hand view (dorsum and palm), orientation (0°, 90°, 180°, and 270°), laterality (left and right), and stimulation condition (left, right and sham).

	Dorsum				Palm			
	0°	90°	180°	270°	0°	90°	180°	270°
Right hand								
Left rTMS	1040	1205	1564	1099	1329	1458	1408	1326
	439	455	596	438	385	519	421	477
Right rTMS	1006	1192	1533	1097	1290	1425	1488	1181
	226	413	667	320	313	512	724	435
Sham	946	1230	1498	1046	1302	1348	1340	1095
	390	371	595	403	373	396	455	287
Left hand								
Left rTMS	1159	1091	1504	1361	1255	1156	1483	1337
	456	467	439	564	446	380	596	458
Right rTMS	1121	1050	1553	1275	1239	1214	1443	1311
	391	270	578	443	341	233	419	295
Sham	1118	1076	1464	1196	1238	1181	1414	1202
	319	261	483	366	399	368	437	242

**Table 3:** Mean accuracy and SD, separately for hand view (dorsum and palm), hand position (lateral and medial), and stimulation condition (left, right, and sham).

	Dorsum		Palm	
	Lateral	Medial	Lateral	Medial
Left rTMS	0.82	0.89	0.80	0.82
	0.22	0.22	0.20	0.20
Right rTMS	0.92	0.89	0.85	0.89
	0.13	0.22	0.16	0.14
Sham	0.94	0.90	0.81	0.87
	0.11	0.18	0.24	0.21

**Table 4:** Mean RTs (msec) and SD, separately for hand view (dorsum and palm), hand position (lateral and medial), and stimulation condition (left, right and sham).

	Dorsum		Palm	
	Lateral	Medial	Lateral	Medial
Left rTMS	1283	1095	1397	1241
	474	445	451	385
Right rTMS	1233	1074	1368	1198
	403	279	365	288
Sham	1213	1061	1275	1138
	325	301	284	295
